# Characterization of mitochondrial genomes in six newly sequenced species and analysis of gene rearrangements across Encyrtidae (Hymenoptera, Chalcidoidea)

**DOI:** 10.3897/zookeys.1285.183757

**Published:** 2026-07-16

**Authors:** Zhi-Peng Chen, Lu-Jie An, Yang-Yi Jia, Hong-Xia Hou, Guo-Hao Zu

**Affiliations:** 1 College of Horticulture and Landscape, Tianjin Agricultural University, Tianjin, 300392, China College of Horticulture and Landscape, Tianjin Agricultural University Tianjin China; 2 College of Chemical Engineering and Biotechnology, Xingtai University, Xingtai, Hebei, 054000, China College of Chemical Engineering and Biotechnology, Xingtai University Xingtai China; 3 Hebei Key Laboratory of Digital Freshwater Aquaculture Technology, Xingtai University, Xingtai, China Hebei Key Laboratory of Digital Freshwater Aquaculture Technology, Xingtai University Xingtai China

**Keywords:** Biological control, Encyrtidae, gene rearrangement, mitochondrial genome, phylogeny, selective pressure

## Abstract

Encyrtidae plays a significant ecological and agricultural role in the biological control of pests. However, the limited mitogenome data and unresolved phylogenetics impede the evolutionary research. We sequenced and annotated the complete mitogenomes of six Encyrtidae species (*Blastothrix speciosa*, *Encyrtus aurantii*, *Lamennaisia ambigua*, *Lamennaisia nobilis*, *Cheiloneurus chinensis*, and *Tassonia gloriae*). All six mitogenomes exhibit the typical circular structure, containing 13 protein-coding genes (PCGs), 22 transfer RNAs (tRNAs), two ribosomal RNAs (rRNAs), and one control region (CR). They show a strong AT bias (81.3–83.8%) and vary in length from 15,397 bp to 17,160 bp. Conserved family-specific gene rearrangements, likely synapomorphic for Encyrtidae, were identified in this study using *Drosophila yakuba* as the ancestral reference species. Species-specific variations, mainly in tRNAs and a few PCGs, appear shaped by positive selection, tRNA structural flexibility, and host adaptation, a pattern that is consistent with the “duplication-random loss” model. Phylogenetic analyses based on the concatenated sequences of 13 PCGs via Maximum Likelihood and Bayesian Inference yielded highly congruent topologies, confirming *Encyrtus* monophyly and the congruence between molecular clustering and morphological taxonomy. This study enriches Encyrtidae mitogenome resources, clarifies the evolutionary rules and gene rearrangements mechanisms, and provides molecular evidence for Encyrtidae systematics and adaptive evolution research.

## Introduction

Chalcidoidea (Hymenoptera) is a highly species-rich group of parasitic wasps, with more than 23,000 described species worldwide (Heraty et al. 2013). In China, 2,129 species across 435 genera and 22 families have been documented, representing a significant component of local insect diversity (UCD Community 2023). As key natural enemies with diverse parasitic strategies, chalcidoids play crucial roles in regulating agricultural and forest pest populations and global integrated pest management (Zhu et al. 2023).

As a core and species-rich family within Chalcidoidea, Encyrtidae holds substantial ecological and agricultural importance. Globally, this family comprises 518 known genera, including 495 valid extant genera (totaling more than 4830 species) and 23 fossil genera (26 species), with a distribution spanning all climatic zones (Simutnik et al. 2023; Wang et al. 2023). Approximately 3710 described species across 455 genera have been reported, reflecting a high taxonomic diversity. A key biological trait of encyrtids is their pronounced host specificity, with many species targeting economically significant pests such as scale insects (Hemiptera: Coccoidea), whiteflies (Hemiptera: Aleyrodidae) (Zhao et al. 2025), and other hemipteran groups. Such host-specific parasitic interactions render encyrtids indispensable for sustainable agriculture and integrated pest management practices worldwide.

Despite their ecological significance, the classification of Encyrtidae has long relied solely on morphological criteria (Noyes and Hayat 1995), with limited auxiliary verification from molecular data. This has led to ongoing debates regarding the monophyly and phylogenetic relationships within the family, particularly concerning the subfamilies Encyrtinae and Tetracneminae (Zhang et al. 2024). Mitochondrial genomes have emerged as core and valuable molecular markers for resolving such systematic ambiguities in insect taxonomy and phylogeny, owing to their conserved gene content, maternal inheritance, moderate evolutionary rate, and rich molecular traits (Krzywinski et al. 2006; Huang et al. 2019; Liu et al. 2024). However, the availability of encyrtid mitogenomes remains scarce: only 1,291 complete Hymenoptera mitogenomes have been deposited in GenBank to date (Sayers et al. 2023), and as of 2026, the database contains more than 1,000 Chalcidoidea mitogenome sequences, among which complete Encyrtidae mitogenomes account for only approximately 12%. This severe data gap hinders accurate phylogenetic reconstructions and comprehensive evolutionary studies of Encyrtidae, though recent studies have begun to address this issue, providing insights into mitogenomic characteristics (e.g., nucleotide composition, gene arrangement, and evolutionary rates of protein-coding genes) and supporting the monophyly of Encyrtidae (Zhang et al. 2024).

A typical insect mitogenome is a closed circular double-stranded DNA molecule, ranging from 14 to 18 kb in length (Zhang et al. 2023), and comprises 13 protein-coding genes (PCGs: *ATP6*, *ATP8*, *COX1*, *COX2*, *COX3*, *CYTB*, *ND1*, *ND2*, *ND3*, *ND4*, *ND4L*, *ND5*, *ND6*), 22 transfer RNA genes (tRNAs), two ribosomal RNA genes (rRNAs: *12S rRNA*, *16S rRNA*), and one non-coding control region (CR)—the latter regulates mitochondrial DNA replication and transcription and is the most variable region in the genome (Liao et al. 2010). Insect mitogenomes generally exhibit a strong AT content bias: more than 70% in most taxa, with Hymenoptera insects showing particular prominence (80–85%) (Liu et al. 2017). This bias directly influences codon usage frequency, typically characterized by high utilization of AT-rich codons such as UUA and AUU (Zhu et al. 2018). Additionally, mitochondrial gene order is highly conserved across most insect taxa, with the ancestral arrangement represented by *D. yakuba* recognized as the primitive pattern of insect mitogenomes; however, frequent gene rearrangements occur in taxa such as Hymenoptera and Thysanoptera, primarily including transposition, inversion, and inverse transposition (Wu et al. 2020). These unique rearrangement patterns provide critical molecular traits for higher-level taxonomic classification and phylogenetic inference, whose evolutionary mechanism is mostly explained by the ‘duplication-random loss model’: redundant copies arise from slipped-strand mispairing during gene duplication, and under selective pressure, these redundant copies gradually accumulate mutations and are lost, ultimately resulting in altered gene order.

The current research on Encyrtidae mitogenomes is further limited by the narrow focus of existing studies, which mostly concentrate on describing mitogenomic characteristics of single species and lack comparative analysis with closely related taxa. For instance, an analysis of *Exoristobia philippinensis* mitogenome identified a rearrangement of *trnW* but no comparative analysis was conducted, making it impossible to reveal taxon-specific patterns of this rearrangement (Feng et al. 2020). In terms of phylogenetic research, findings are constrained by insufficient sample representativeness and limited molecular markers (Zhang et al. 2021). Most studies include only 3–5 genera of Encyrtidae, failing to reflect the overall evolutionary relationships within the family (Zhou et al. 2021). Furthermore, phylogenetic trees are mostly constructed based on *COX1* or partial protein-coding genes (PCGs), which hinders the resolution of phylogenetic conflicts at deep nodes (Cameron 2025). For example, nuclear gene data suggest a close phylogenetic relationship between Encyrtidae and Aphelinidae, whereas mitogenomic data support a sister-group relationship between Encyrtidae and Eulophidae (Peters et al. 2018). This contradiction highlights the necessity of multi-gene concatenated analyses and expanded sample coverage. Additionally, the evolutionary driving mechanisms underlying gene rearrangements in Encyrtidae remain unclear. Existing studies hypothesize that parasitic species exhibit a higher degree of gene rearrangement than phytophagous species, yet quantitative evidence is lacking (Ghosh et al. 2024). The association between selective pressure and gene rearrangement has also not been explored: due to adaptation to the defense mechanisms of different hosts, PCGs in Encyrtidae species may experience stronger positive selection. However, whether this selective pressure promotes gene rearrangement still requires verification by comparing the Ka/Ks ratios across different taxa (San Jose et al. 2023).

To address the aforementioned research gaps, the present study selected six representative species of Encyrtidae—*Blastothrix speciosa*, *Encyrtus aurantii*, *Lamennaisia ambigua*, *Lamennaisia nobilis*, *Cheiloneurus chinensis*, and *Tassonia gloriae*—for mitogenome sequencing. These taxa represent distinct genera and taxonomic positions within Encyrtidae, most of which are morphologically well-defined but lack available molecular data, particularly complete mitogenomes, in public databases to date. The inclusion of these species can effectively improve the phylogenetic resolution of Encyrtidae, clarify intergeneric and intrageneric boundaries, and test the monophyly of key lineages within the family, thereby filling critical gaps in the mitogenomic and phylogenetic research of the superfamily Chalcidoidea. Using optimized DNA extraction and high-throughput sequencing technologies (Ruiz-Mena et al. 2024), we obtained complete mitogenome sequences and analyzed their structural characteristics. To support subsequent phylogenetic and comparative analyses, we further integrated mitogenomic data of eleven closely related Encyrtidae species and one Eulophidae species, all retrieved from GenBank (NCBI, Bethesda, MD, USA). Using these integrated data, we constructed a nucleotide sequence matrix of the 13 protein-coding genes (PCGs) and employed maximum likelihood (ML) and Bayesian inference (BI) methods to reconstruct phylogenetic trees—with the goal of clarifying genus-level phylogenetic relationships within Encyrtidae (Zhang et al. 2019). Meanwhile, we compared the mitochondrial gene arrangement patterns across the 11 species. Combined with analyses of host preference and selective pressure on PCGs (assessed via Ka/KS ratios), we further explored the patterns of gene rearrangement in Encyrtidae and their underlying evolutionary driving mechanisms.

## Materials and methods

### Sample collection and sequencing

Herein, six species within Encyrtidae were newly sequenced and assembled (detailed information is shown in Table 1). All specimens were stored in 99% ethanol at −40 °C before species identification and DNA extraction. To compare the mitochondrial characters of the six newly sequenced species and also for the phylogenetic analyses, we downloaded eleven mitochondrial genomes of Encyrtidae from GenBank. The taxonomist Guohao Zu identified all species based on morphological characteristics.

**Table 1.** Detailed information on newly sequenced species in this study.

Species	Location	Date	Accession Number
*Blastothrix speciosa*	Lijiang, Yunnan	July 24, 2022	OR189373
*Encyrtus aurantii*	Guangzhou, Guangdong	October 16, 2022	OR120384
*Lamennaisia ambigua*	Chuxiong, Yunnan	July 24, 2022	OR189375
*Lamennaisia nobilis*	Chuxiong, Yunnan	July 24, 2022	NC_061411
*Cheiloneurus chinensis*	Jinghai, Tianjin	July 18, 2018	NC_084192
*Tassonia gloriae*	Guangzhou, Guangdong	October 16, 2022	NC_082112

Total genomic DNA was extracted from tissue samples of six species using the DNeasy Blood & Tissue Kit (Qiagen, Hilden, Germany). Following extraction, the total DNA was quantified for concentration and assessed for quality using a microanalyzer and 1% agarose gel electrophoresis. Raw sequencing data for six species of Encyrtidae were generated on the Illumina HiSeq 6000 Sequencing Platform (Illumina, San Diego, CA, USA) with a 350-bp insert length and 150-bp paired-end sequencing mode. All sequencing work was performed by Novogene Co., Ltd. (Beijing, China).

### Assembly, annotation, and composition analyses

After the raw sequencing data were obtained from the sequencing company, quality control was performed using fastp v. 0.23.4 (Chen et al. 2018) to obtain clean data with a quality value > 30. The mitogenome was then assembled using two independent tools, namely MitoZ v. 3.6 (Meng et al. 2019) and GetOrganelle v. 1.7.7.0 (Jin et al. 2020). For annotation, we used reference sequences from related Eulophidae species available in GenBank and performed the annotation via the Galaxy platform (Afgan et al. 2016). The secondary structures of transfer RNAs (tRNAs) were predicted using the Galaxy platform and visualized using VARNA v. 3.9 (Darty et al. 2009), while the complete mitogenome map was generated using the CGview Server. For sequence characterization, nucleotide composition, and relative synonymous codon usage (RSCU) of PCGs were analyzed using MEGA v. 11.0.13 (Tamura et al. 2021). Nucleotide skews (AT-skew and GC-skew) were calculated according to the following formulas: AT-skew = (A−T)/(A+T) and GC-skew = (G−C)/(G+C) (Perna and Kocher 1995).

### Phylogenetic analysis

Phylogenetic analysis was performed using 18 mitochondrial genomes, representing two families of Chalcidoidea, including 17 species from Encyrtidae and one from Eulophidae. Phylogenetic trees were reconstructed using both maximum likelihood (ML) and Bayesian inference (BI) methods. Individual sequence alignment for each PCG was performed using the online MAFFT v. 7 service with the L-INS-i strategy. Aligned sequences were trimmed using GBlocks (Talavera and Castresana 2007) and subsequently concatenated into a combined PCG dataset using PhyloSuite v. 1.2.3 (Zhang et al. 2020). The optimal nucleotide substitution model was selected based on the Bayesian information criterion (BIC) using ModelFinder v. 2.2.0 (Kalyaanamoorthy et al. 2017). For BI analysis, MrBayes v. 3.2.7a was used with four Markov chains and two independent runs of 2,000,000 generations each, with sampling every 1,000 generations. The first 25% of trees were discarded as burn-in. Convergence was confirmed when the average standard deviation of split frequencies was < 0.01 and the potential scale reduction factor (PSRF) approached 1.0. ML analysis was implemented in IQ-TREE v. 2.2.0 (Nguyen et al. 2015) with 1,000 bootstrap replicates under the standard bootstrap approximation.

## Results

### Mitogenomic organization

To investigate the mitogenomic characteristics of Encyrtidae, we performed high-throughput sequencing on six species: *B. speciosa*, *E. aurantii*, *L. ambigua*, *L. nobilis*, *C. chinensis*, and *T. gloriae*. Approximately 6 Gb of raw sequencing data was generated for each species, and all newly assembled mitogenomes exhibited a complete circular structure. These mitogenomic sequences have been deposited in the GenBank database, with accession numbers listed in Table 1. In addition, detailed information on the mitogenomic architecture of the six Encyrtidae species, all of which were curated via manual verification and annotation, is provided in Suppl. material 1.

**Figure 1.**
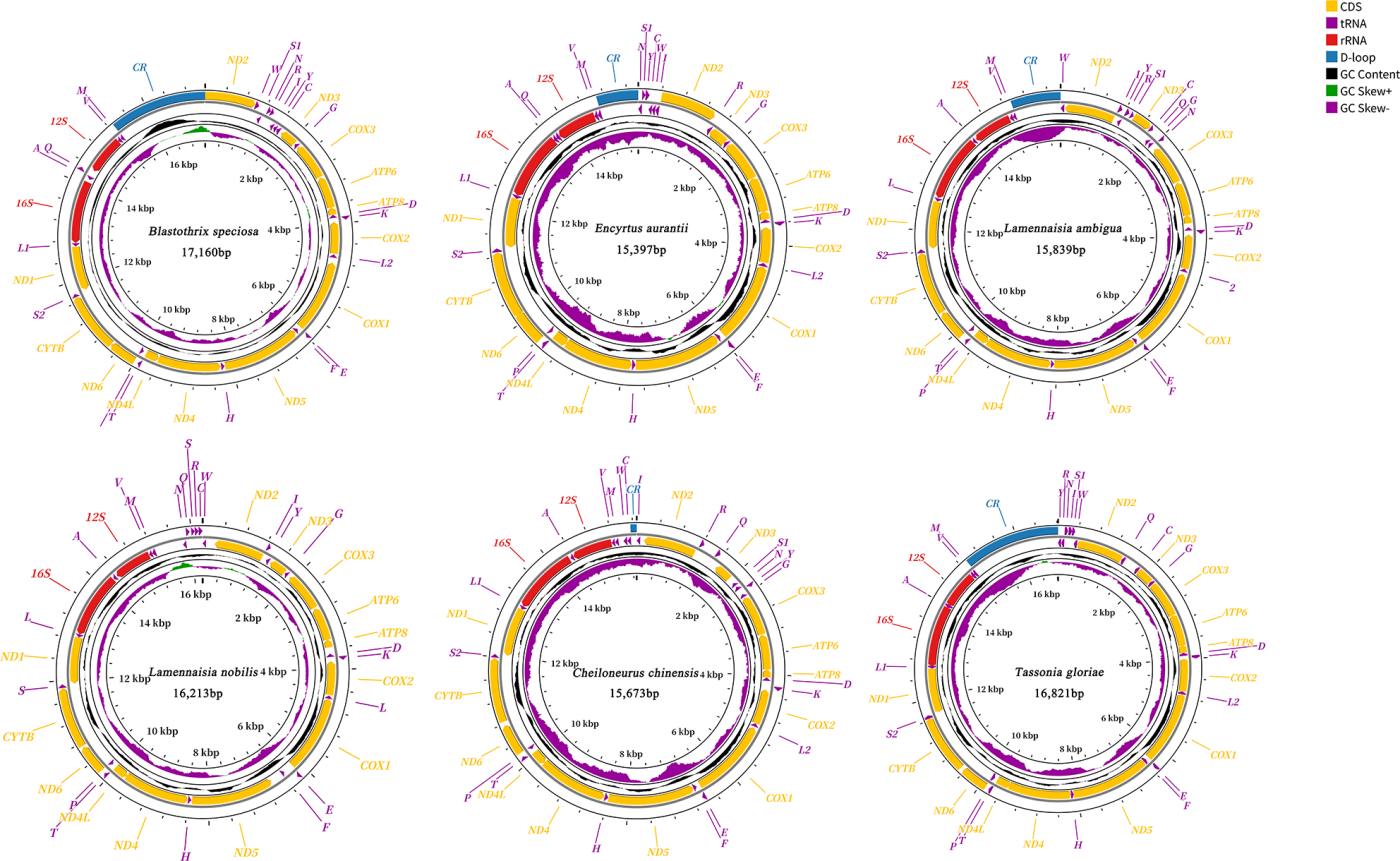
Mitogenome maps of newly sequenced species. The arrow indicates the direction of gene transcription. Normative abbreviations are used to represent protein-coding genes (PCGs) and ribosomal RNA genes (rRNAs), while transfer RNA genes (tRNAs) are denoted by single-letter abbreviations. The GC content of the complete mitogenomes is shown in the second circle. The GC-skew of the complete mitogenomes is shown in the third circle. The innermost circle shows the lengths of the complete mitogenomes of each species (ranging from 15,397 bp to 17,160 bp).

Structural annotation revealed that each mitogenome contains one control region (CR), two ribosomal RNA genes (rRNAs), 22 transfer RNA genes (tRNAs), and 13 protein-coding genes (PCGs). The total length of the newly assembled mitogenomes ranges from 15,397 bp to 17,160 bp (Fig. 1).

In terms of base composition, most of the newly sequenced mitogenomes show a positive AT skew, with values ranging from 0.084 (*B. speciosa*) to 0.133 (*C. chinensis*). In contrast, all newly reported mitogenomes exhibit a negative GC skew, with values between −0.283 (*C. chinensis*) to −0.152 (*B. speciosa*). Additionally, the overall AT content varies from 81.30% (*T. gloriae*) to 83.78% (*L. ambigua*) (Suppl. material 2).

### Protein–coding genes, codon usage, and RSCU

The 13 protein-coding genes (PCGs) identified in the six newly sequenced Encyrtidae species exhibited conserved gene content, consistent with the typical mitochondrial genomic architecture of Chalcidoidea insects. The concatenated PCG sequences ranged in length from 11,061 bp (*L. ambigua*) to 11,127 bp (*E. aurantii*).

The length of control region (CR) varied considerably among the six Encyrtidae species, ranging from 177 bp in *C. chinensis* to 1882 bp in *T. gloriae*. In Encyrtidae species, all PCGs utilize ATN (ATA, ATT, ATG) as initiation codons. Across the 13 PCGs of the six newly sequenced Encyrtidae species, ATA functions as the initiation codon for *ND2* and *ND4L* in *B. speciosa*, *ND3* and *ND4L* in *E. aurantii*, *ATP8* and *ND1* in *L. ambigua*, *ND2*/*ND3*/*ND1* in *C. chinensis*, and *ND5* in *T. gloriae*. All remaining PCGs employ either ATT or ATG as their initiation codons. Among the three initiation codon variants (ATA, ATT, ATG) identified, ATA displays the lowest utilization frequency, whereas ATT and ATG are both prevalent across the surveyed PCGs. For termination codons, all PCGs use TAN (TAA, TAG), TA, or a single T residue as termination signals. Among the six species, TAA represents the most prevalent termination codon, followed by TAG; TA is less frequent, and a single T residue is the rarest. Notably, *E. aurantii* uniquely exhibits four instances of a single T residue serving as the termination codon (Suppl. material 1).

The AT content of the PCGs ranged from 78.8% in *T. gloriae* to 83.1% in *B. speciosa*. For all six species, the AT-skew of PCGs exhibited a range of –0.183 in *T. gloriae* to 0.165 in *L. ambigua* (Suppl. material 2).

**Figure 2.**
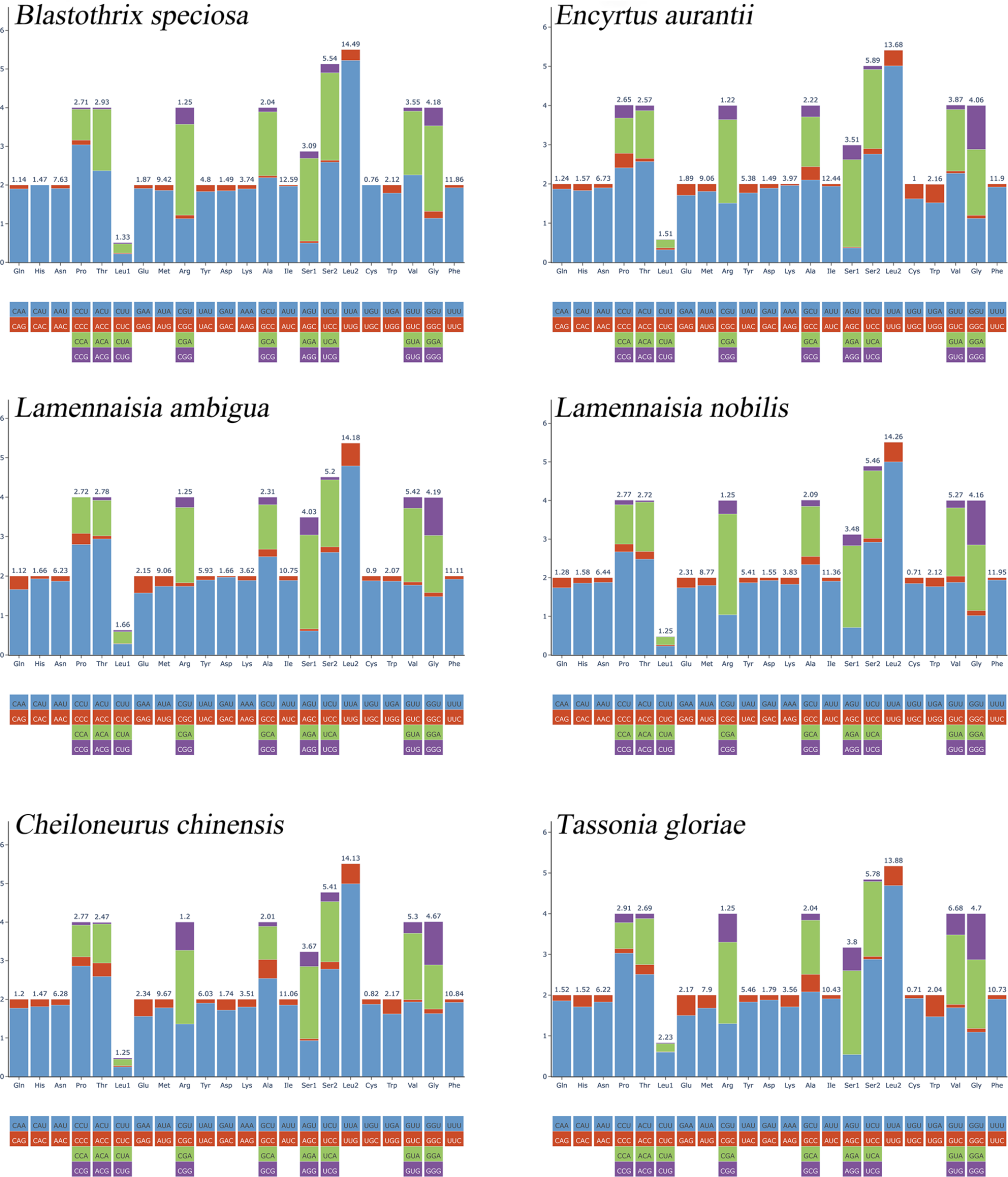
Relative synonymous codon usage (RSCU) of mitochondrial protein-coding genes (PCGs) in six newly sequenced Encyrtidae species.

The codon UUA (Leu2) was the most commonly used in the mitogenomes of all six Encyrtidae species (Fig. 2). The mitochondrial PCGs of the six species exhibited an obvious bias towards A and T. In *B. speciosa*, *E. aurantii*, *L. ambigua*, *L. nobilis*, and *C. chinensis*, the three most frequently used codons (RSCU > 2.0) were UUA (Leu2), AUU (Ile), and UUU (Phe). In *T. gloriae*, the three most used codons (RSCU > 2.0) were UUA (Leu2), UCA (Ser2), and CGA (Arg). In the six Encyrtidae species, mitochondrial PCGs showed a clear preference for A and U at the third codon position. This was reflected in the predominant usage of NNA and NNU codons, with the RSCU values of these codons accounting for 68–75% of total synonymous codon usage.

This consistent codon usage bias is directly correlated with the strong AT bias of Encyrtidae mitogenomes, and may be associated with the efficiency of mitochondrial replication and transcription.

### Gene rearrangement

**Figure 3.**
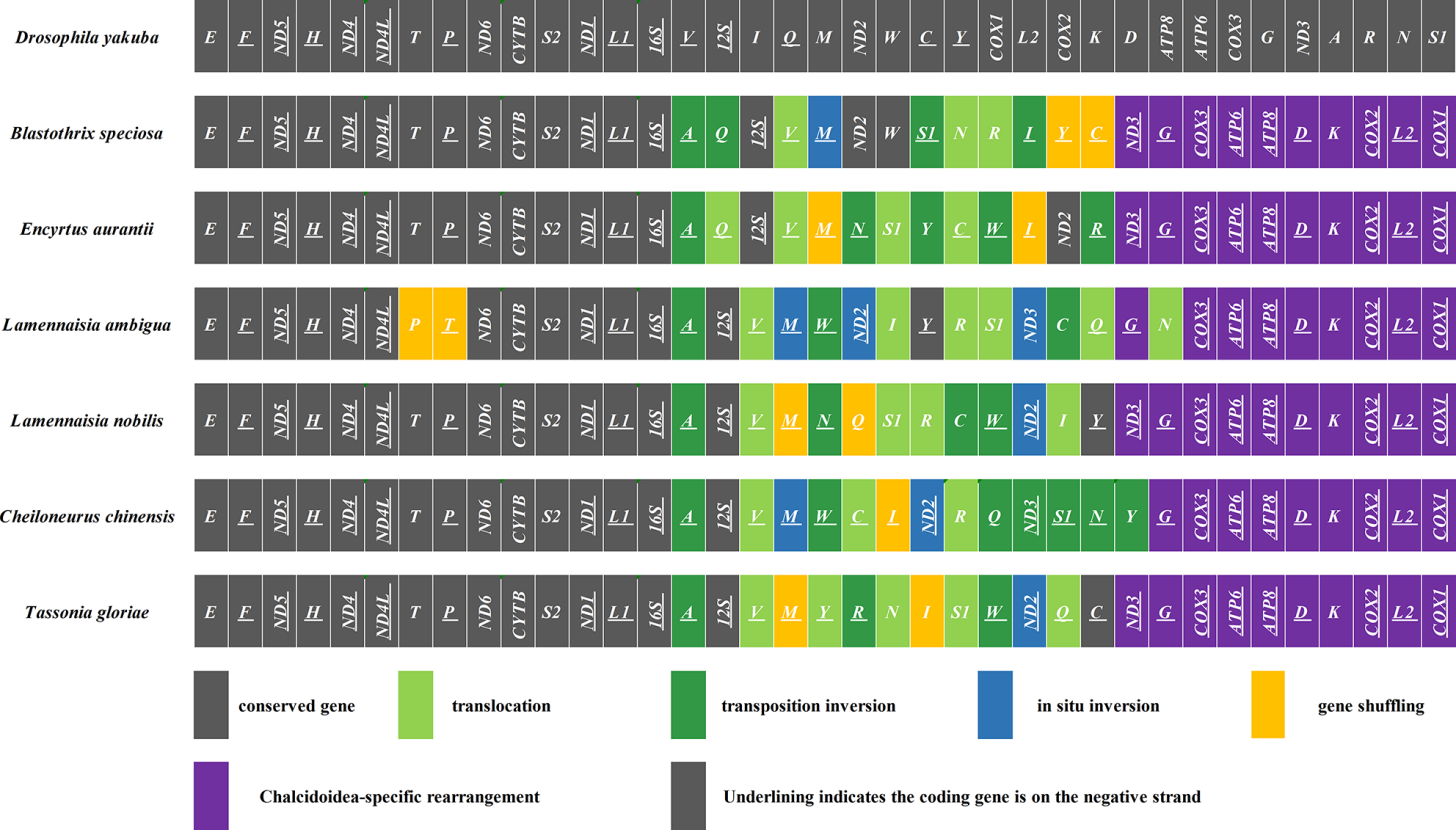
Gene arrangements of newly sequenced mitochondrial genomes. Mitochondrial genomes are depicted as linearized gene arrays, where each block represents a single gene (Transfer RNA genes (*trns*) are abbreviated using their corresponding amino acid single-letter codes; for example, *trnA* is denoted as “A”). The *D. yakuba* genome serves as the ancestral gene arrangement reference for insects. Color coding indicates different genome features: gray blocks represent conserved genes; green blocks denote translocation; dark green blocks indicate transposition inversion; blue blocks mark in situ inversion; yellow blocks represent gene shuffling; and purple blocks denote Chalcidoidea-specific rearrangement. Underlined blocks indicate genes encoded on the negative strand.

Using the ancestral insect mitochondrial genome (*D. yakuba*) as a reference, we analyzed the mitochondrial gene rearrangements in six Encyrtidae species (*B. speciosa*, *E. aurantii*, *L. ambigua*, *L. nobilis*, *C. chinensis*, and *T. gloriae*). The results revealed both family-level conserved patterns and species-specific variations, as detailed below. All species retained a conserved core set of genes, which included six protein-coding genes (*ND5*, *ND4*, *ND4L*, *ND6*, *CYTB*, *ND1*), two ribosomal RNA genes (*16S*, *12S*), and five transfer RNA genes (*trnE*, *trnF*, *trnH*, *trnS2*, *trnL1*). This conservation aligns with the overall conserved characteristics of Encyrtidae (Fig. 3).

At the family-level, the shared rearrangement patterns encompass the specific translocation of *trnA* downstream of *16S*, as well as the translocation of *trnV* downstream of *12S*; both translocation events are consistently observed across all six sequenced Encyrtidae species. Furthermore, the gene cluster *COX1-trnL2-COX2-trnK-trnD-ATP8-ATP6-COX3-trnG-ND3* exhibits a Chalcidoidea-specific rearrangement signature, which is largely conserved among the six Encyrtidae species analyzed. Specifically, all genes within this cluster undergo directional reversal relative to the ancestral reference sequence (*D. yakuba*), with the exception of *trnK*, which retains its original orientation.

**Figure 4.**
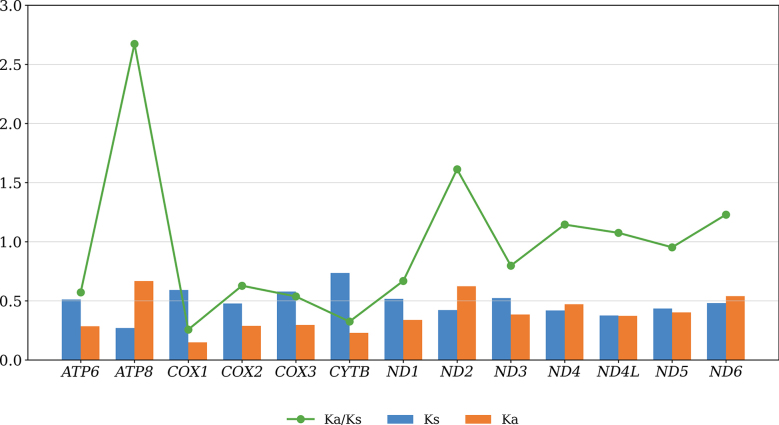
Evolutionary rates of protein-coding genes in the mitochondrial genomes of Encyrtidae. Blue and orange bars indicate the average synonymous (Ks) and non-synonymous (Ka) substitution rates, respectively. The green line represents the mean Ka/Ks ratio, calculated based on 17 Encyrtidae species with *Chouioia cunea* as the outgroup.

To assess the selective constraints on mitochondrial protein-coding genes (PCGs) and their correlation with gene rearrangements in Encyrtidae, we calculated the mean ratio of non-synonymous to synonymous substitution rates (Ka/Ks) for each of the 13 PCGs across 17 ingroup Encyrtidae species, with the outgroup taxon as the reference (Fig. 4).

Most PCGs were under strong purifying selection (mean Ka/Ks < 1). Among them, *COX1* had the lowest Ka/Ks value (0.26), followed by *CYTB* (0.32). Both genes belong to the core conserved PCGs with no rearrangement events detected in this study, indicating that purifying selection maintains the structural and functional integrity of key oxidative phosphorylation genes.

Five PCGs showed signatures of positive selection with mean Ka/Ks > 1, ranked from highest to lowest: *ATP8* (2.67), *ND2* (1.61), *ND6* (1.23), *ND4* (1.14), and *ND4L* (1.07). All these positively selected genes are located within or adjacent to the identified mitochondrial rearrangement hotspots, including the *COX1*–*ND3* cluster and NADH dehydrogenase gene cluster with family- or species-specific structural variations. Notably, *ND3*, which harbored a unique in situ inversion in *Lamennaisia ambigua*, had a moderate Ka/Ks value (0.80) close to 1, suggesting relaxed purifying selection that may be associated with the occurrence of lineage-specific gene rearrangements.

### Phylogenetic relationships

**Figure 5.**
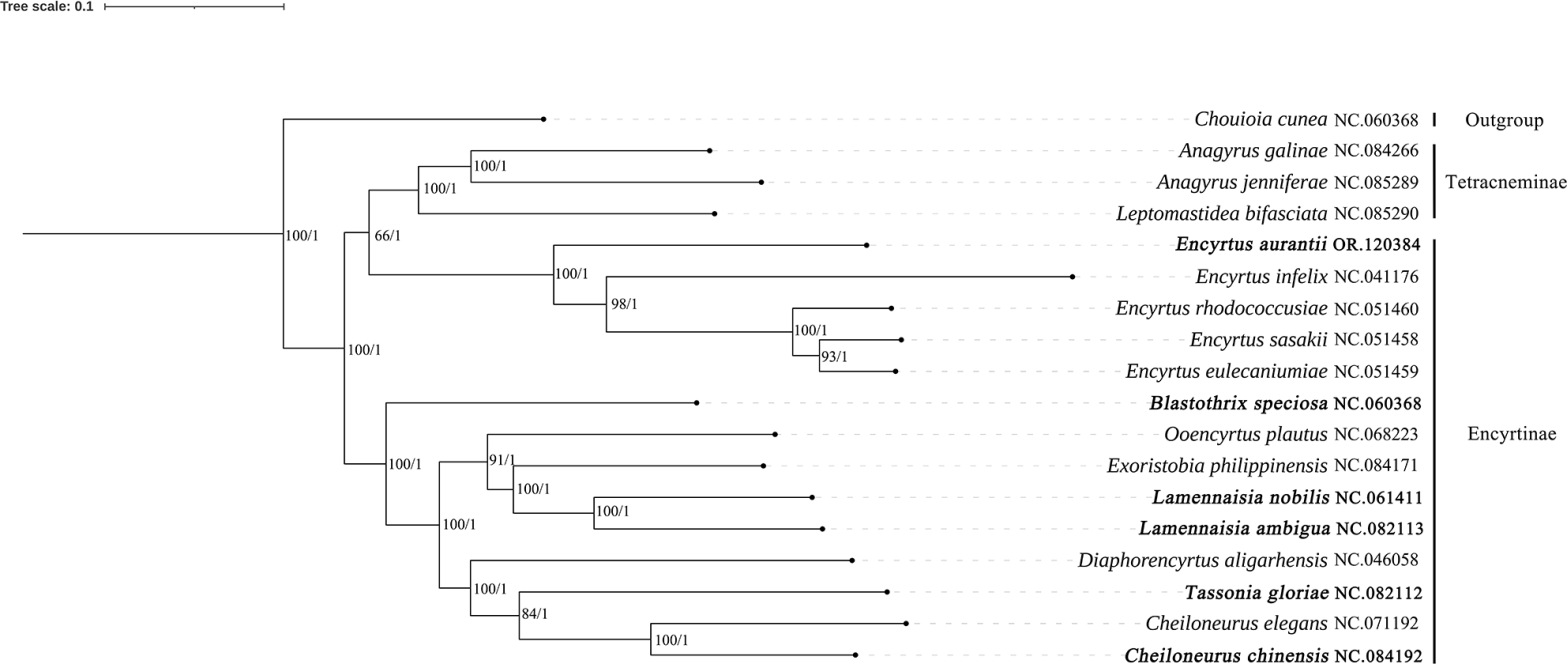
The phylogenetic tree was constructed based on 13 PCGs by Bayesian inference and maximum likelihood methods. The number at each node indicate the posterior probability and bootstrap values resulting from the analyses (ML on the left and BI on the right).

Phylogenetic relationships among the six target species, analyzed within a broader Encyrtidae dataset, were inferred using both Maximum Likelihood (ML) and Bayesian Inference (BI) methods. Branch support was assessed using ML bootstrap (BS) value and BI posterior probability (PP). All nodes in the resulting topology received maximum Bayesian posterior probability support (PP = 1.0), with ML bootstrap (BS) values ranging from 66% to 100% (Fig. 5). The analysis was based on the concatenated nucleotide sequences of 13 PCGs. The resolved intrafamilial phylogenetic topology within Encyrtidae is structured as follows: (((*Anagyrus galinae* + *Anagyrus jenniferae*) + *Leptomastidea bifasciata*) + (*Encyrtus aurantii* + (*Encyrtus infelix* + (*Encyrtus rhodococcusiae* + (*Encyrtus sasakii* + *Encyrtus eulecaniumiae*))))) + (Blastothrix speciosa + ((*Ooencyrtus plautus* + (*Exoristobia philippinensis* + (*Lamennaisia nobilis* + *Lamennaisia ambigua*))) + (*Diaphorencyrtus aligarhensis* + (*Tassonia gloriae* + (*Cheiloneurus elegans* + *Cheiloneurus chinensis*))))).

Within this topology, *A. galinae* and *A. jenniferae* form a sister clade, which subsequently clusters with *L. bifasciata*. The genus *Encyrtus* is recovered as a well-supported monophyletic group, with *E. aurantii* occupying a basal position relative to the other four congeneric species (*E. eulecaniumiae*, *E. sasakii*, *E. rhodococcusiae*, and *E. infelix*). *C. chinensis* and *C. elegans* constitute a close lineage, and are resolved as the sister group to *T. gloriae*. Meanwhile, *L. ambigua* and *L. nobilis* formed a monophyletic clade, consistent with their congeneric taxonomic status established based on morphological characteristics.

## Discussion

### Mitochondrial genome structure

The six newly sequenced Encyrtidae species possess circular mitochondrial genomes with typical insect mitogenomic composition, comprising 13 PCGs, 22 tRNAs, two rRNAs, and one CR. The lengths of mitogenomes range from 15,397 bp in *B. speciosa* to 17,160 bp in *T. gloriae*. This variation in size is largely attributable to the CR. *T. gloriae* exhibits the longest CR (1,882 bp), whereas *C. chinensis* has the shortest (177 bp), a pattern consistent with the high variability of CR in hymenopteran mitogenomes (Bai et al. 2023). Nucleotide composition analysis reveals a strong AT bias (81.30–83.78%) in the six species, consistent with the typical AT-rich feature of hymenopteran mitogenomes. This bias is further reflected in their codon usage: UUA (Leu2) is the most frequently used codon across all species, and AT-rich codons such as AUU and UUU dominate the relative synonymous codon usage (RSCU > 2.0) in most taxa. Furthermore, a clear preference for NNA and NNU codons is observed at the third codon position, accounting for 68–75% of total synonymous codon usage (Li et al. 2024). These distinct synonymous codon usage biases are driven primarily by the extreme AT bias of Encyrtidae mitogenomes. The preference for AT-rich codons and A/U at the third codon position is an adaptive strategy that optimizes the efficiency of mitochondrial replication, transcription, and translation, while reducing replication errors (Wolstenholme 1992; Li et al. 2024). This conserved pattern across our six newly sequenced species reflects a stable family-level evolutionary trait of Encyrtidae under extreme AT content, shaped by compositional constraints, mitochondrial expression machinery, and purifying selection. In terms of nucleotide skews, most mitogenomes show a positive AT-skew (0.084–0.133) and a negative GC-skew (−0.283 to −0.152), indicating a slight preference for adenine (A) over thymine (T) and cytosine (C) over guanine (G). This pattern is also observed in other Chalcidoidea families and likely related to the replication and transcription mechanisms of insect mitogenomes (Huang et al. 2023). The PCGs of the six species are conserved in length, ranging from 11,061–11,127 bp, and predominantly use ATN as the start codon—specifically ATG, ATA, ATT, and in rare cases ATC) (Zhang et al. 2020). No alternative start codons such as TTG were detected, suggesting a conserved initiation codon pattern within Encyrtidae. Overall, the structural characteristics of the newly obtained mitogenomes, such as genomic composition, AT bias, codon usage, and nucleotide skew patterns, are consistent with previously reported data from Encyrtidae and other Chalcidoidea lineages. These results confirm the evolutionary conservation of mitogenomic organization within this group, while also revealing lineage-specific variations, including differences in CR length, that may be associated with adaptive evolution.

### Mitochondrial gene rearrangements

Mitochondrial genome rearrangement analyses of six Encyrtidae species (with *D. yakuba* as the ancestral reference) reveal a hierarchical conservation-divergence pattern in gene organization. At the gene content level, all species retain a conserved core set: six protein-coding genes (*ND5*, *ND4*, *ND4L*, *ND6*, *CYTB*, *ND1*), two rRNA genes (*16S*, *12S*), and five tRNA genes (*trnE*, *trnF*, *trnH*, *trnS2*, *trnL1*), reflecting Encyrtidae’s inherent mitogenomic stability. At the rearrangement level, two universal translocations—*trnA* downstream of *16S* and *trnV* downstream of *12S*—act as diagnostic family markers; additionally, the *COX1-trnL2-COX2-trnK-trnD-ATP8-ATP6-COX3-trnG-ND3* cluster carries a largely conserved Chalcidoidea-specific signature, defined by directional reversal of all genes (except *trnK*) relative to *D. yakuba*, underscoring phylogenetic affinity to the superfamily.

To further verify the family specificity and synapomorphic status of the aforementioned rearrangements, we compared the mitochondrial gene order of Encyrtidae with two closely related families in Chalcidoidea (Eulophidae and Aphelinidae). Mitogenomic data showed that Eulophidae exhibited heterogeneous rearrangement patterns at the genus or subfamily level, with no conserved family-specific gene order. *Tamarixia radiata* only displayed limited tRNA gene rearrangements with a highly conserved protein-coding gene (PCG) block (Du et al. 2019), *Tetrastichus howardi* merely harbored tRNA cluster shuffling without consistent family-level gene order changes (Tang et al. 2021), while *Chouioia cunea* possessed a genus-specific large-scale gene inversion, rather than a synapomorphy for the entire family (Tang et al. 2021). Aphelinidae showed a much lower rearrangement frequency, with rearrangements limited to local tRNA shuffling and highly conserved PCG order. *Encarsia* species only presented scattered tRNA gene rearrangements with no consistent family-level traits (Polaszek et al. 2025), and three *Aphelinus* species retained a completely conserved PCG order relative to the ancestral insect type, with tRNA rearrangements only detected near the control region. In contrast, the two characteristic translocations (*trnA* translocated to the downstream of *16S rRNA*, *trnV* translocated to the downstream of *12S rRNA*) were universally conserved in all analyzed Encyrtidae species, but completely absent in Eulophidae and Aphelinidae. This distinct lineage-specific divergence confirms that these conserved rearrangements represent synapomorphies of Encyrtidae, providing valuable evidence for the higher classification and phylogenetic analysis of Chalcidoidea.

Rare lineage-specific variations, such as the inversion of *ND3* in *L. ambigua*, shuffling between *trnQ* and *trnM* in *L. nobilis*, and shuffling of *trnC* and *trnY* in *C. chinensis*, arise from interrelated evolutionary drivers. First, positive selection contributes to these variations by enhancing genomic flexibility for adaptive rearrangements, as evidenced by higher Ka/Ks ratios in Encyrtidae protein-coding genes (PCGs) relative to non-parasitic taxa; this pattern aligns with the first documented case of positive selection in Encyrtidae, where the *ND2* gene of *Ooencyrtus plautus* exhibited a Ka/Ks ratio of 1.1 (Xing et al. 2022). Second, the structural lability of tRNA genes—attributed to their small molecular size and modular organization—increases their susceptibility to shuffling or inversion, a trait well characterized in Chalcidoidea parasitoids (Chen et al. 2018). Third, host-specific adaptation plays a role, as congeneric Encyrtidae species sharing similar host associations often display analogous rearrangement patterns; this observation supports broader findings that gene rearrangement divergence within Chalcidoidea correlates with differentiation in host utilization (Beckenbach 2012). The “duplication-random loss” model further provides a molecular mechanism underlying these variations: slipped-strand mispairing during mitochondrial DNA replication generates redundant gene copies, and subsequent selective loss of these copies reshapes gene order—a process widely validated as a key driver of insect mitochondrial genome rearrangements (Li et al. 2024). Collectively, these factors illustrate that Encyrtidae mitochondrial genomes maintain a balance between conservation, which ensures genomic stability, and divergence, which facilitates adaptive evolution, ultimately shaping the evolutionary trajectory of mitochondrial organization in this family (Song et al. 2022).

Selective pressure analysis supported the correlation between evolutionary constraints and gene rearrangements in Encyrtidae. Conserved genes under strong purifying selection (e.g., *COX1*, *CYTB*) were structurally stable without rearrangements. Positively selected genes (*ATP8*, *ND2*, *ND6*, *ND4*, *ND4L*) were distributed in rearrangement hotspots, and *ND3* with relaxed purifying selection underwent a unique inversion. This pattern indicates that reduced selective constraints promote lineage-specific gene rearrangements in Encyrtidae.

### Phylogenetic relationship analyses

The family Encyrtidae is taxonomically classified into two subfamilies, Tetracneminae and Encyrtinae (Simutnik et al. 2023), and all six newly sequenced Encyrtidae species in this study were assigned to Encyrtinae, establishing a clear taxonomic basis for interpreting their phylogenetic affinities. Phylogenetic analyses using Maximum Likelihood (ML) and Bayesian Inference (BI) methods yielded congruent topological patterns: at the subfamilial level, the six Encyrtinae species displayed closer phylogenetic relationships (supported by consistent ML/BI topologies) while showing distant affinities to Anagyrinae taxa, including the genus *Anagyrus* and species *Leptomastidea bifasciata*. This topological divergence aligns with the established taxonomic framework of Encyrtidae and further validates the effectiveness of mitochondrial protein-coding genes (PCGs) – the molecular marker used in this study – for resolving subfamily-level phylogenetic relationships within the family.

At the generic level, strong phylogenetic clustering of congeneric species was observed, which reinforces the reliability of current generic delimitations. Specifically, the newly sequenced *Encyrtus aurantii* clustered robustly with other *Encyrtus* species to form a well-supported monophyletic clade, directly reflecting close genetic affinity among congeneric taxa and confirming previous morphology-based taxonomic hypotheses for *Encyrtus*. Similarly, *Leptomastidea ambigua* and *Leptomastidea nobilis* formed a cohesive subclade that further clustered with *Exoristobia philippinensis* into a monophyletic group (Chi et al. 2024), suggesting a shared evolutionary history among these species and providing molecular evidence for their taxonomic association within Encyrtinae. Notably, *Tassonia gloriae* formed a well-supported monophyletic clade with two *Cheiloneurus* species (including the newly sequenced *Cheiloneurus chinensis*); this strongly supported yet unexpected topology (consistent across ML and BI trees) highlights the need for further investigation into potential morphological or ecological synapomorphies uniting *Tassonia* and *Cheiloneurus*, as current taxonomic treatments of these genera have relied primarily on morphological traits. Collectively, these results not only confirm the taxonomic placement of the six newly sequenced species within Encyrtinae, but also provide novel molecular insights into generic and subfamilial relationships of Encyrtidae, emphasizing the value of integrating molecular data with traditional morphology to advance Encyrtidae systematics.

## Conclusions

This study sequenced and annotated the complete mitochondrial genomes of six Encyrtidae species: *B. speciosa*, *E. aurantii*, *L. ambigua*, *L. nobilis*, *C. chinensis*, and *T. gloriae*. These mitogenomes exhibit the typical circular structure of insect mitogenomes, consisting of 13 PCGs, 22 tRNAs, two rRNAs, and one CR. They also show a pronounced AT bias (81.3–83.8%) and vary in length from 15,397 to 17,160 bp, a range largely attributable to differences in the length of the CR. Using *D. yakuba* as the ancestral reference species, we analyzed mitochondrial gene rearrangement patterns in the six Encyrtidae species and identified three conserved family-specific traits: the ectopic inversion of *trnA* downstream of *16S*, the transposition of *trnV* downstream of *12S*, and the complete reversal of the *COX1-trnL2-COX2-trnK-trnD-ATP8-ATP6-COX3-trnG-ND3* gene cluster, in which all components except *trnK* are inverted. These rearrangements likely represent synapomorphic characteristics of Encyrtidae. Comparative analysis with closely related Chalcidoidea families further confirmed that the three conserved mitochondrial gene rearrangements are synapomorphic for Encyrtidae, providing robust molecular markers for the systematics of this family. Species-specific variations were predominantly observed in tRNAs and a few PCGs. Examples include the in situ inversion of *ND3* in *L. ambigua*, gene shuffling of *trnQ*/*trnM* in *L. nobilis* and *trnC*/*trnY* in *C. chinensis*. These rearrangement events are fully explained by the “duplication-random loss” model—the most widely validated mechanism for insect mitogenome evolution, which attributes gene order changes to replication slippage-induced gene duplication and subsequent random loss of redundant copies, with selective pressure further filtering the retained adaptive gene variations. Phylogenetic analyses based on concatenated sequences of 13 PCGs, conducted using both ML and BI methods, produced highly congruent topologies. The results confirm the monophyly of *Encyrtus* and are consistent with morphological taxonomy at the genus level. Overall, this study enriches the mitogenome resources of Encyrtidae, clarifies the evolutionary patterns of gene rearrangements in the family, and elucidates their underlying driving mechanisms. The results reveal both conserved family-specific patterns and lineage-specific variations, these findings provide robust molecular evidence for the systematics and lay a foundation for subsequent studies on adaptive evolution and potential applications in biological control.

## Additional information

### Artificial Intelligence (AI) use

The authors accept full responsibility for the content of the manuscript, including the disclosure of any use of AI.

No AI tools were used in the preparation of this manuscript.

### Author ORCIDs

Z.-P. Chen https://orcid.org/0009-0006-9947-5074

L.-J. An https://orcid.org/0009-0004-6987-1573

Y.-Y. Jia https://orcid.org/0009-0001-5535-1452

H.-X. Hou https://orcid.org/0000-0002-0203-8809

G.-H. Zu https://orcid.org/0000-0002-9892-2171

### Data availability

Data presented in this study are openly available in the NCBI repository with accession numbers: OR189373, OR120384, OR189375, NC_061411, NC_084192, NC_082112.

## Supplementary materials

Supplementary material 1 Gene organization of the mitochondrial genomes of newly sequenced species. Data type xls https://binary.pensoft.net/file/1717666This dataset is made available under the Open Database License (http://opendatacommons.org/licenses/odbl/1.0/). The Open Database License (ODbL) is a license agreement intended to allow users to freely share, modify, and use this Dataset while maintaining this same freedom for others, provided that the original source and author(s) are credited. Zhi-Peng Chen

Supplementary material 2 Nucleotidae features of the mitochondrial genome of newly sequenced species. Data type xlsx https://binary.pensoft.net/file/1717668This dataset is made available under the Open Database License (http://opendatacommons.org/licenses/odbl/1.0/). The Open Database License (ODbL) is a license agreement intended to allow users to freely share, modify, and use this Dataset while maintaining this same freedom for others, provided that the original source and author(s) are credited. Zhi-Peng Chen
